# Validation of nucleolar protein 4 as a novel methylated tumor suppressor gene in head and neck cancer

**DOI:** 10.3892/or.2013.2927

**Published:** 2013-12-16

**Authors:** SEMRA DEMOKAN, ALICE Y. CHUANG, KAVITA M. PATTANI, DAVID SIDRANSKY, WAYNE KOCH, JOSEPH A. CALIFANO

**Affiliations:** 1 Department of Basic Oncology, Oncology Institute, Istanbul University, Capa, Istanbul 34093, Turkey; 2 Department of Otolaryngology-Head and Neck Surgery, Johns Hopkins University, School of Medicine, Baltimore, MD, USA; 3 Department of Dermatology, Johns Hopkins University, School of Medicine, Baltimore, MD, USA; 4 Milton J. Dance Head and Neck Center, Greater Baltimore Medical Center, Baltimore, MD, USA

**Keywords:** nucleolar protein 4, methylation, head and neck cancer, candidate gene approach

## Abstract

Methylation of CpG islands in the promoter region of genes acts as a significant mechanism of epigenetic gene silencing in head and neck cancer. In the present study, we assessed the association of epigenetic alterations of a panel of 12 genes [nucleolar protein 4 (*NOL4)*, iroquois homeobox 1 (*IRX1)*, *SLC5A8*, *LRRC3B*, *FUSSEL18*, *EBF3*, *GBX2*, *HMX2*, *SEPT9*, *ALX3*, *SOCS3* and *LHX6*] with head and neck squamous cell carcinoma (HNSCC) via a candidate gene approach. After the initial screening of methylated CpG islands on the promoter regions by bisulfite sequencing using salivary rinse samples, only two genes had methylated CpG dinucleotides on their promoter regions in tumor samples and absence of methylated CpGs were found in normal salivary rinse samples after bisulfite modification and bisulfite sequencing. We then performed real-time quantitative methylation-specific PCR (QMSP) on 16 salivary rinse and 14 normal mucosal samples from healthy subjects and 33 HNSCC tumor samples for the two genes selected. After validation with QMSP, one gene, *NOL4*, was highly methylated (91%) in tumor samples and unmethylated in normal salivary rinses and minimally methylated in normal mucosal samples demonstrating cancer-specific methylation in HNSCC tissues. Although the *IRX1* gene was observed as methylated in normal mucosal and salivary rinse samples, the methylation values of these normal samples were very low (<10%). In conclusion, we identified *NOL4* as a highly specific promoter methylated gene associated with HNSCC. *IRX1* may have potential as a biomarker for HNSCC and should be assessed in a larger cohort.

## Introduction

Head and neck cancer, which is the sixth most common cancer in the world among human malignant disorders, is an aggressive and life-threatening disease with poor prognosis, morbidity and high mortality in advanced disease. Survival rates have not improved significantly for patients with head and neck squamous cell carcinoma (HNSCC) in the past 30 years despite active clinical and basic research addressing this issue. More than 40,000 new cases of HNSCC are diagnosed in the United States each year, with a mortality rate of 12,000 in the USA annually (1). Treatment for HNSCC includes surgical resection, chemotherapy and radiation therapy; however, approximately 50% of all patients have advanced disease at the time of diagnosis often requiring use of all three treatment modalities. Cancer-specific molecular biomarkers, which have the ability to warn the clinicians in the earlier stage before the disease advances, or to provide insight regarding the prognosis of the disease or outcome of the patients, are required. In addition, it is important to develop new methods that provide sensitive and reliable biomarkers of HNSCC for detection, treatment response and prognosis.

Epigenetic alterations are a recent attractive phenomenon of human cancer, with the activation of proto-oncogenes and inactivation of tumor suppressor genes, either through hypomethylation or hypermethylation in the promoter regions of the genes, respectively (2). Transcriptional silencing of tumor suppressor genes by means of promoter hypermethylation plays an important role in head and neck carcinogenesis (3). Methylation of the CpG islands in the promoter regions of tumor suppressor genes is frequently observed with reduced gene expression (4,5).

From a previous study using the gene expression profiling via oligonucleotide microarray-based approach to discover the new cancer-specific methylated genes (6), in the present study, we evaluated the hypermethylation of 10 genes [nucleolar protein 4 (*NOL4*), iroquois homeobox 1 (*IRX1*), sodium-coupled monocarboxylate transporter 1 (*SLC5A8*), leucine rich repeat containing 3B (*LRRC3B*), functional smad-suppressing element on chromosome 18 (*FUSSEL18*), early B-cell factor 3 (*EBF3*), gastrulation brain homeobox 2 (*GBX2*), H6 family homeobox 2 (*HMX2*), septin 9 (*SEPT9*), ALX homeobox 3 (*ALX3)*] identified by Restriction Landmark Genomic Scanning (RLGS) in previous studies (7,8), and by personal communication with Bennett *et al* (7,8), and two other genes [suppressor of cytokine signaling 3 (*SOCS3*) and LIM homeobox 6 (*LHX6*)] selected from the literature via candidate gene approach (9–14). *IRX1*, *SLC5A8*, *FUSSEL18*, *EBF3*, *GBX2*, *HMX2*, *SEPT9* and *ALX3* genes showed tumor suppressor activity in previous cancer studies (15–19) and were involved in transforming growth factor (TGF) signaling pathway which has a high frequency of alteration in HNSCC (15,20–23). To measure methylation levels, real-time quantitative methylation-specific PCR (QMSP) was performed to provide an objective, robust and rapid assessment of promoter methylation status (24–27).

## Materials and methods

### Tissue samples

Following institutional review board approval and after obtaining appropriate informed consent, the HNSCC patients and control population from healthy subjects enrolled in a community screening study were recruited from the Johns Hopkins School of Medicine, Department of Otolaryngology-Head and Neck Surgery. Mucosal samples and salivary rinses from healthy population and HNSCC tissue samples were collected. In the present study, salivary rinses were obtained by brushing oral cavity and oropharyngeal surfaces with an exfoliating brush followed by rinse and gargle with 20 ml normal saline solution. The brush was gently agitated to release the obtained material into saline. After centrifugation, the supernatant was discarded and DNA was isolated from the pellet. Tumors were snap frozen and microdissected on a cryostat to ≥75% purity. DNA from 16 salivary rinse samples from non-cancer individuals were analyzed as a control, to investigate the normal promoter methylation status of two newly identified candidate genes, *IRX1* and *NOL4*. The methylation status of these genes was analyzed in 33 fresh tumor samples from patients with head and neck cancer and 14 normal mucosa samples from healthy individuals.

### DNA extraction and bisulfite treatment

DNA was isolated as previously described (28). In brief, DNA was obtained by phenol/chloroform extraction after overnight incubation with proteinase K (Boehringer-Mannheim, Germany) at 48°C. DNA from tumor and control samples was subjected to bisulfite treatment using EpiTect Bisulfite modification kit (Qiagen, Valencia, CA, USA) as per the manufacturer’s protocol.

### Bisulfite sequencing

The bisulfite sequence analysis was performed to determine the methylation status in the promoter regions of 12 genes. Bisulfite-treated DNA was amplified for the 5′ region that included at least a portion of the CpG island within 1–2 kb of the first exon of the genes. The promoter regions of the genes were found from the database of the University of California, Santa Cruz (UCSC) (http://genome.ucsc.edu/). Primer sequences were determined by MethPrimer program (29) showing the CpG islands in the promoter regions of 12 genes for bisulfite sequencing (Table I). A thousand base pair region of the genes’ promoters and some part of the first exon were sequenced by the specific primers producing 400–500 bp PCR fragments. The primers for bisulfite sequencing were designed to hybridize to regions in the promoter without CpG dinucleotides. PCR products were gel-purified using the QIAquick Gel Extraction kit (Qiagen) according to the manufacturer’s instructions. Each amplified DNA sample was sequenced by the Applied Biosystems 3700 DNA Analyzer using nested, forward or reverse primers and BD terminator dye (Applied Biosystems, Foster City, CA, USA).

### Quantitative methylation-specific PCR

Primer and probe sequences were determined by MethPrimer program showing the CpG islands in the promoter regions of two genes selected after bisulfite sequencing (Table II). To determine if the methylated genes in tumor samples were cancer-specific, we investigated promoter methylation in 16 normal saliva, 14 age-matched normal mucosa from healthy individuals that were analyzed as a control, to investigate the normal promoter methylation status of two newly identified candidate genes (*NOL4*, *IRX1*) and in 33 HNSCC tumor samples by QMSP. Lymphocytes obtained from a healthy individual were *in vitro* methylated using excess SssI methyltransferase (New England Biolabs Inc., Beverly, MA, USA) to generate completely methylated DNA that was used as a positive control standard. To quantitate the relative percent of methylation, we computed the ratio between the QMSP values of the gene of interest relative to an internal control, *ACTB* (gene of interest/reference gene ×100) (30). Fluorogenic PCR was carried out in a reaction volume of 20 μl consisting of 600 nM of each primer; 200 nM of probe; 0.6 U of platinum Taq polymerase (Invitrogen, Carlsbad, CA, USA); 200 μM of each dATP, dCTP, dGTP and dTTP; 1X ROX Dye reference and 1X buffer [16.6 mM of ammonium sulfate; 67 mM of Trizma (Sigma, St. Louis, MO, USA); 6.7 mM of magnesium chloride; 10 mM of mercaptoethanol and 0.1% dimethylsulfoxide]. Thirty nanograms of bisulfite treated DNA were used in each real-time QMSP reaction. Amplifications were carried out in 384-well plates in a 7900 Sequence Detector system (Perkin-Elmer Applied Biosystems, Norwalk, CT, USA) and were analyzed by SDS 2.3 (sequence detector system) (Applied Biosystems). Each reaction was performed in triplicate.

## Results

### Clinicopathological characteristics of control subjects and patients with HNSCC

Table III describes the demographic parameters of the sample populations used in the present study. The mean age of normal mucosal samples was 43.4 years (range, 24–65). Tobacco users were observed as 42%. Both normal mucosal and tumor samples had a similar male and Caucasian predominance. Smoking rate was 78% and alcohol consumption was 69%. Tumor samples (n=33) were obtained from patients with stage I (7.4%), stage II (22%), stage III (26%) and stage IV (44%) lesions. These were from primary tumors of the oral cavity (n=9), oropharynx (n=7), hypopharynx (n=2), larynx (n=8), maxillary sinus (n=2), nasal floor (n=1), salivary gland (n=1) and unknown primary/neck (n=3). The male and Caucasian prevalence was smaller in the normal salivary rinse samples and tobacco users were found as 31.25%. The ages of individuals from which the normal salivary rinse was obtained were slightly lower than the population of head and neck cancer patients, mean ages 53.06 years (range, 33–83) and 61.4 years (range, 36–88), respectively. Due to our small cohort, we did not perform any statistics between the clinical parameters and QMSP results of tumor and normal populations.

### Genes specifically methylated in HNSCC tumors

In the present study, we investigated methylation of the 12 gene promoters by bisulfite modification and QMSP. In the initial screening of the methylated CpG islands, bisulfite sequencing was performed by using four HNSCC cell lines (JHU-06, JHU-022, JHU-022B and JHU-028), eight normal salivary rinses and eight HNSCC samples. Only two genes, *NOL4* (NM_003787) and *IRX1* (NM_024337) (http://www.genenames.org), had methylated CpG dinucleotides on their promoter regions in tumor samples but absence of methylated CpGs was found in normal salivary rinse samples (Table IV). We investigated the methylation frequency in a larger cohort of normal salivary rinses, mucosal and HNSCC specimens, in order to find a biomarker candidate.

**Figure 1:**
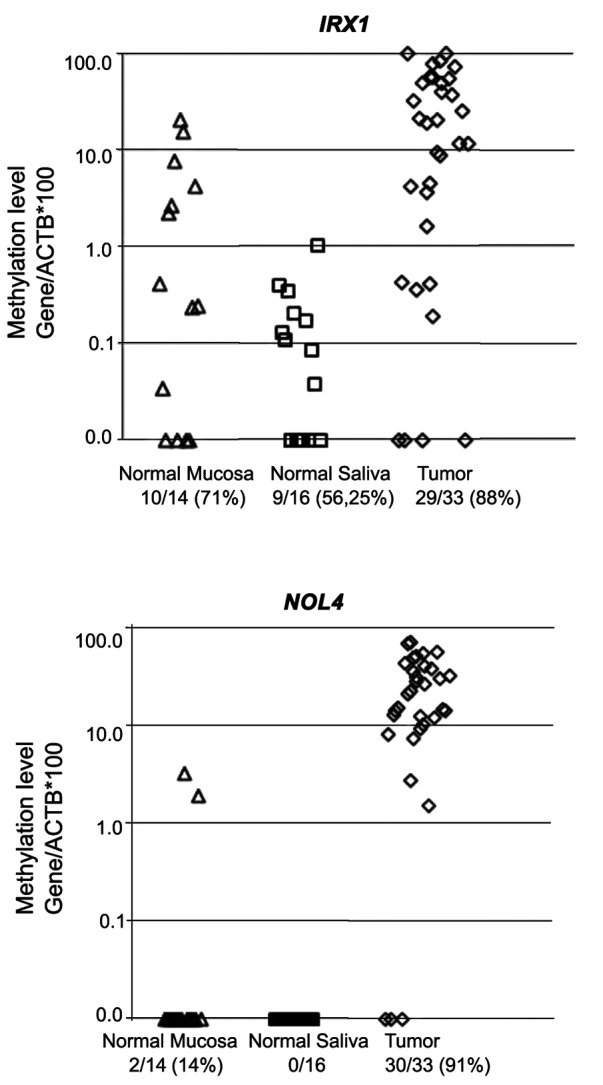
Methylation frequency of two candidate genes *IRX1* and *NOL4* in HNSCC tumors, normal mucosal and salivary rinse samples. Scatter plots of QMSP analysis of candidate gene promoters. *NOL4*, nucleolar protein 4; *IRX1*, iroquois homeobox 1; HNSCC, head and neck squamous cell carcinoma; QMSP, real-time quantitative methylation-specific PCR.

In the second stage of the present study, we performed QMSP on 16 normal salivary rinse and 14 normal mucosal samples from healthy individuals and 33 HNSCC tumor samples for two selected genes. The *NOL4* gene showed no methylation (0/16) in normal salivary rinses and two out of 14 (14%) mucosal samples were minimally methylated between 1 and 3% methylation values, whereas the methylation rate was 91% (30/33) on the promoter region of the *NOL4* gene in HNSCC tumor samples showing high methylation values are 79% (26/33) of the patients between 10 and 100%. The *IRX1* gene [10/14 (71%), 9/16 (56.25%) and 29/33 (88%)] demonstrated varying degrees of methylation on their promoter regions in normal mucosa, normal salivary rinses and HNSCC tumor samples, respectively (Fig. 1). Although normal salivary methylation rate was observed as high, *IRX1* methylation values were <1% in normal salivary rinses and were mostly (8/14, 57%) between 0.1 and 10% in normal mucosal samples indicating a very strong marker which may define HNSCC tumor tissues as a potential biomarker (Fig. 1).

## Discussion

In the present study, we investigated methylation status of the promoter regions of 12 genes by bisulfite modification, bisulfite sequencing and QMSP techniques. Only two of them (*IRX1* and *NOL4*) showed methylation in their CpG islands located on promoter region of these genes, indicating a characteristic of a biomarker molecule. *NOL4* gene encoding a nucleolar protein which is expressed predominantly in brain and testis, was identified by Ueki *et al* (31) and there is only one study showing high methylation status of 20 patients with cervical cancer by MethyLight assays (32) in concordance with our results.

The *IRX1* gene is a member of the iroquois homeobox gene family and plays a role during pattern formation of vertebrate embryos. In published literature, there are only four studies investigating the effects of epigenetically silencing *IRX1* gene, in the patients with gastric cancer (33) and the studies of Bennett *et al* with HNSCC (7,8,34). Bennett *et al* (7) reported an association between the HNSCC and hypermethylation of *IRX1* in accordance with our results, and four other genes (*FUSSEL18*, *EBF3*, *SLC5A8* and *SEPT9*), but not *SLC5A8;* we did not find any methylated CpG on the promoter regions of the last five genes. In two other studies, Bennett *et al* also showed the association between *IRX1* methylation and recurrence and between four other genes and clinicopathological parameters such as HPV status, alcohol and tobacco usage (8) and they investigated the interactions with some molecules of the TGF-β pathway, and their transcriptional inactivation by methylation in HNSCC caused the decrease in apoptosis and differentiation, and increased proliferation (34). In the present study, we only observed the methylation of the *IRX1* gene which was the one of the five frequently methylated genes identified by Restriction Landmark Genomic Scanning (RLGS) method in metastatic HNSCC samples compared to primary tumors. Mass array methylation (7) and COBRA (8) analysis were used in fresh/paraffin-embeded HNSCC tumors and matched normal mucosa samples in the previous studies of Bennett *et al* (7,8). We screened the methylated CpG islands on the promoter regions of these genes by bisulfite sequencing using frozen HNSCC tumors, HNSCC cell lines and normal salivary rinses in order to select the genes that have the methylated CpG islands on their promoter regions in tumor samples but no methylation in normal mucosal and normal salivary rinse samples. Then, the methylation levels were measured by QMSP technique in 33 HNSCC tumors, 16 salivary rinses and 14 normal mucosa as reported in our previous study (35). Our aim was to find a cancer-specific biomarker and to detect the tumor tissues in salivary rinses of the patients earlier and to screen the normal population. In addition, in a previous study (34), tonsillar carcinoma samples were used, whereas we quantified the methylation in the primary tumors of the oral cavity, oropharynx, hypopharynx, larynx, maxillary sinus, nasal floor and unknown primary/neck. Therefore, discrepant observations between these studies by Bennett *et al* (7,8,34) and our data may be due to confounding factors including anatomic site, methodology and sampling method. Therefore, the four other genes described by Bennett *et al* (7,8), may not be good biomarker candidates in salivary rinse samples despite being differentially methylated in primary tumor samples. The present study is the first to investigate the methylation levels of *NOL4* gene and to show high methylation in the patients with HNSCC by QMSP technique.

In addition, it would be helpful to increase sample size to facilitate a more precise determination of accuracy of these biomarkers in detection of the disease and to evaluate the association of the results with the clinical parameters of the disease. These newly identified silenced genes here remain to be tested in saliva, serum or plasma samples from HNSCC patients in a larger sample size.

## Figures and Tables

**Table I:** Primer sequences of 12 selected genes for bisulfite sequencing.

Gene name	5′ Primer sequence 3′	Primer sequence
*LRRC3B*	TAAAGAGAGGGGAAAGATTTTTGTT	AATCAATTTCCCCTACAATTCTAAAA
	GGAAAATTGAATTTTATTTTTTTT	AAAATATTAACTCCCTCTACTACTCTC
	AATAGGAGAAAGAATGGGGTTATAGTT	TAACCTTACAAAAAAAACAAACAAAA
*NOL4*	GGAAGTTTTGAATGGAGTAATTGTT	CAAATACATTTTAAATAAATTCCAACC
	GTTTGGGGTATTATAATTTATTTTGTAGAA	CTCTCCTTCCTCCTAAATCCTACTT
	TAGGATTTAGGAGGAAGGAGAGATT	ACACCATTCTAACCCAAAAAAACTA
	GTTGGGATGGTTTTGGTTATAAA	CACCTATTCACCCTAAACTCATAAAA
*FUSSEL18*	TTATTTAATTATTTGAGATTAGAATTA	AATAAATTCCTAAAAAACCTAAAACCTTAT
	GGTTTTAGGTTTTTTAGGAATTTAT	TCACCTAACCCACCTAATTAAATTTAA
*EBF3*	TTTTAGGATAAGTTGTAGTTTTTTGTATTT	ATTTAACCCCTTAATACCTCCCTAC
	GTAGGGAGGTATTAAGGGGTTAAAT	CACAAAACTCAACCCTCTCTCCC
	GGGAGAGAGGGTTGAGTTTTGTG	CCCAAACATAAAAACTACTAAC
*GBX2*	GAGGGGTAGGATTTTGTTTTTAATT	AACCTTAAAACCCTACAACCTTATC
	GAGGTTAGTTTGGGTGGAAAG	ATAAAACATAAACATAAAATAACC
	GGTAGGTAAAATGTGAATGAGAAAGAGGAG	ATAAAACATAAACATAAAATAACC
*IRX1*	TGGGTGAAGAGAAAGTTTTTTTT	AAACATCTTTAACAAAAATACACCC
	TTTTGTTAAAGATGTTTTTTGGAGG	TACTTTAATTAACATCCCCTTAAAC
*HMX2*	GTTTTGTTATTAGTTTTTTATTTTTTTT	AACCTCATCCCTATCACAAATTCTA
	GGAATTTGAATTTAGATTTTTTG	TTAAACCCCTTAAACCCTTCTCT
	GAGAGAAGGGTTTAAGGGGTTTA	TACAACAAACAAACAATAAAAAAAA
	GGAGGATGGTGGAGTAGTTTGTATA	TCTAACCAAAAACAACCAAAACTAAA
*SLC5A8*	GTGGATTGTTTATTTAGGATTAGATGG	AACCTTCATATAACACATACATACTTAACA
	GTGTTAAGTATGTATGTGTTATATGAAGGT	AAAAACTAAAACCTCCAACTACTTCC
	GATTGTTGAATTGGAAAGTTAAAATTTA	CCTCAAACCCAAATATAAAACCTC
*SEPT9*	GGAAGATGTTTTTTTTGTTAAGGAG	TCAATCTATACTACTCCCCAAAACC
	TTGGGGAGTAGTATAGATTGAAAAGT	TTAAACTTCACCTCAAAAATTCATT
	GGATGAATAGTGGGGAATAGTATTG	CCAAAAAAAACCCTAAAAAATCAC
*ALX3*	GTGGGTTTTTAGATATTTGGGTTATT	CAAACAAACAAACCTTAAACTACAATTT
	ATTTTTAATAGTTTTTTTTATTGTG	CTAACTTAATACTAAAACCATCCAC
	TTTGAGTTGTTTGGGATTGG	CTCTAAAAAATAAAACTCCAAAAACC
*SOCS3*	GAGAGTATTTGGTTTAATTTATA	CTTCCCCTTCCCCTTTTCCC
	TGTAGTTTTGGGTTTTTTTTT	CAACTTCTCATTCACATTTCC
	GATTTGGATTTTTTGTTT	CTCCTCCTTCCTACCTAATC
*LHX6*	GTAGATGGTATGGTTATGGGT	ACCTCCCTAACTACTACC
	AGGAGGATAAGGAGGAGGGAG	CTCATACTTCCAATACATAAACC
	GTTTTTGTAGTAGTTTTTGT	CCAACATTTACATAATATATTCC
	GGTTTATGTATTGGAAGTATGAG	AAAAAAAAACACCCTCCAACC
	GGTTGGAGGGTGTTTTTTTTT	AATTTTTTCTCTCTCCACC

*LRRC3B*, leucine rich repeat containing 3B; *NOL4*, nucleolar protein 4; *FUSSEL18*, functional smad-suppressing element on chromosome 18; *EBF3*, early B-cell factor 3; *GBX2*, gastrulation brain homeobox 2; *IRX1*, iroquois homeobox 1; *HMX2*, H6 family homeobox 2; *SLC5A8*, sodium-coupled monocarboxylate transporter 1; *SEPT9*, septin 9; *ALX3*, ALX homeobox 3; *SOCS3*, suppressor of cytokine signaling 3; *LHX6*, LIM homeobox 6.

**Table II:** Primers and probe sequences of 2 selected genes for validation by QMSP.

Gene name	Probe sequence	5′ Primer sequence	3′ Primer sequence	Temp (°C)	bp
*NOL4*	GGGGAGGCGGCGTTGCGTTTTAT	TTTTCGGGGTTTAAAGGCGTTG	AAATAATCCCTAAACGCCTCGC	60	178
*IRX1*	AGTAGTTGGTCGGGTCGGTACGG	GGGGATATATTTCGGTCGCGA	TCCCGCGAACACGTAATACC	60	198

QMSP, real-time quantitative methylation-specific PCR; *NOL4*, nucleolar protein 4; *IRX1*, iroquois homeobox 1.

**Table III:** QMSP results and demographics of the patients with HNSCC.

Tumor samples	*IRX1*	*NOL4*	Age (years)	Gender	Race	Smoking	Alcohol	Tumor anatomic site	Overall stage
1	N	N	67	M	C	Yes	Yes	Nasal floor	2
2	Y	Y	57	M	C	Yes	Yes	Larynx	2
3	N	N	61	M	C	No	No	Neck	3
4	Y	Y	60	F	C	Yes	Yes	Larynx	4
5	Y	Y	55	M	A	Yes	Yes	Larynx	2
6	Y	Y	54	M	C	No	Yes	Oropharynx	4
7	Y	N	64	M	A	Yes	Yes	Hypopharynx	3
8	Y	Y	55	F	A	Yes	No	Oral cavity	1
9	N	Y	80	M	C	Yes	No	Oral cavity	NA
10	Y	Y	54	F	C	No	Oral	cavity	4
11	Y	Y	62	M	C	Yes	Yes	Oropharynx	4
12	Y	Y	72	M	C	Yes	Yes	Hypopharynx	3
13	Y	Y	42	M	C	Yes	No	Larynx	2
14	Y	Y	66	M	C	Yes	No	Oropharynx	4
15	Y	Y	74	M	C	Yes	Larynx	2	
16	Y	Y	58	M	A	Yes	Yes	Oropharynx	4
17	Y	Y	56	F	C	Yes	Yes	Oral cavity	2
18	Y	Y	43	M	C	Yes	Yes	Oropharynx	4
19	Y	Y	68	M	C	Yes	Yes	Oropharynx	4
20	Y	Y	63	M	A	Yes		Oral cavity	NA
21	Y	Y	64	F	C	No	Yes	Oral cavity	3
22	N	Y	88	M	C	Yes	Yes	Oral cavity	NA
23	Y	Y	42	M	C	Yes	No	Oral cavity	3
24	Y	Y	51	M	C	Yes	Yes	Larynx	4
25	Y	Y	80	M	C	No	Yes	Neck	NA
26	Y	Y	58	M	C	Yes	Yes	Larynx	3
27	Y	Y	71	M	C	Yes	Yes	Neck	NA
28	Y	Y	48	M	C	Yes	Yes	Oropharynx	4
29	Y	Y	61	M	C	Yes	No	Maxillary sinus	1
30	Y	Y	77	M	C	No	No	Salivary gland	NA
31	Y	Y	67	M	C	Larynx	3		
32	Y	Y	36	M	C	Yes	No	Oral cavity	4
33	Y	Y	74	F	C	No	Yes	Maxillary sinus	4

M, male; F, female; C, Caucasian; A, African; Y, methylated; N, unmethylated; QMSP, real-time quantitative methylation-specific PCR; HNSCC, head and neck squamous cell carcinoma; *IRX1*, iroquois homeobox 1; *NOL4*, nucleolar protein 4; NA, not available.

**Table IV:** Information regarding candidate tumor suppressor genes and the bisulfite sequencing results.

Gene ref. ID	Chromosomal location	Candidate TSGs	Normal salivary rinses n (%)	HNSCC tissue n (%)	HNSCC cell lines n (%)
NM_052953	chr3:26,664,300–26,752,265	*LRRC3B*	1/3 (33)	3/4 (75)	4/4 (100)
**NM_003787**	**chr18:31,431,070–31,803,446**	***NOL 4***	**0/6 (0)**	**6/6 (100)**	**4/4 (100)**
NM_001037802	chr18:44,757,495–44,775,554	*FUSSEL18*	5/8 (62.5)	4/7 (57)	3/4 (75)
NM_001005463	chr10:131,633,547–131,762,091	*EBF3*	2/4 (50)	1/3 (33)	3/4 (75)
NM_001485	chr2:237,074,307–237,076,652	*GBX2*	3/5 (60)	4/6 (67)	4/4 (100)
**NM_024337**	**chr5:3,596,168–3,601,517**	***IRX1***	**0/6 (0)**	**6/6 (100)**	**4/4 (100)**
NM_005519	chr10:124,907,638–124,910,188	*HMX2*	2/4 (50)	1/3 (33)	4/4 (100)
NM_145913	chr12:101,549,994–101,604,016	*SLC5A8*	3/5 (60)	2/3 (67)	2/2 (100)
NM_006640	chr17:75,315,597–75,496,678	*SEPT9*	4/7 (57)	2/3 (67)	2/4 (50)
NM_006492	chr1:110,602,997–110,613,322	*ALX3*	1/3 (33)	0/3 (0)	3/4 (75)
NM_003955	chr17:76,352,859–76,356,158	*SOCS3*	0/8 (0)	0/8 (0)	1/2 (50)
NM_014368	chr9:124,964,858–124,991,019	*LHX6*	0/8 (0)	0/8 (0)	1/1 (100)

HNSCC, head and neck squamous cell carcinoma; *LRRC3B*, leucine rich repeat containing 3B; *NOL4*, nucleolar protein 4; *FUSSEL18*, functional smad-suppressing element on chromosome 18; *EBF3*, early B-cell factor 3; *GBX2*, gastrulation brain homeobox 2; *IRX1*, iroquois homeobox 1; *HMX2*, H6 family homeobox 2; *SLC5A8*, sodium-coupled monocarboxylate transporter 1; *SEPT9*, septin 9; *ALX3*, ALX homeobox 3; *SOCS3*, suppressor of cytokine signaling 3; *LHX6*, LIM homeobox 6.
